# Does Alexithymia Affect Memory for a Crime? The Relationship Between Alexithymia, Executive Functions, and Memories

**DOI:** 10.3389/fpsyg.2021.669778

**Published:** 2021-06-30

**Authors:** Fabiana Battista, Tiziana Lanciano, Antonietta Curci

**Affiliations:** ^1^Department of Education, Psychology, Communication, University of Bari “Aldo Moro”, Bari, Italy; ^2^Leuven Institute of Criminology, Catholic University of Leuven, Leuven, Belgium

**Keywords:** eyewitness, alexythimia, memory distortions, forgetting, executive functions

## Abstract

Prior studies on alexithymia and memory have found a negative association between the two constructs, especially when emotional memories are considered. Moreover, it has been demonstrated that also the executive functioning (EF) of the individuals influences this relationship. Thus, the goal of this study is to verify whether alexithymia can influence the memory accuracy for a violent crime in people with different levels of EF resources in terms of both correct details and memory distortions (i.e., omissions and commissions) reported. We assessed the alexithymia and EF resources of individuals and showed participants a video of a violent crime (i.e., murder). We then asked participants to testify about the content of the video by imagining to be witnesses of the crime. A memory test was run on two moments in time: immediately after the video presentation and after 10 days. Findings demonstrated that alexithymia influences the recall of the event both in proneness to report correct details and memory distortions of the participants (i.e., omissions and commissions). Additionally, we found a contribution of EF resources in this relationship. The findings provide new information for legal professionals on memory functioning.

## Introduction

Alexithymia has been defined as a personality construct specifically characterized by difficulties in identifying feelings (DIFs), difficulties in describing feelings (DDFs), and externally oriented thinking (EOT) (e.g., Sifneos, 1973; Nemiah, 1977; Bagby et al., 1994a,b; Taylor, 2000; Kooiman et al., 2002; Vermeulen and Luminet, 2009). In the 1960's, alexithymia was conceived as a personality disorder (e.g., Kooiman et al., 2002) due to a strong link to psychosomatic dysfunction, such as anxiety, depression, posttraumatic stress disorder, abuse of alcohol and drugs, and eating disorders (e.g., Zeitlan and Mcnally, 1993; Petterson, 2004; Li et al., 2015; Brady et al., 2016). Some studies have also found that alexithymia might mediate the relationship between maltreatment during childhood and psychopathologies (e.g., Paivio and McCulloch, 2004; Serafini et al., 2017). However, many recent studies have shown that alexithymia generally occurs also in individuals with no declared diseases (e.g., Franz et al., 2008; Donges and Suslow, 2015); thus, it is to be considered as a personality variable rather than a disorder (e.g., Donges and Suslow, 2015). In other words, alexithymia is a personality characteristic that is present in all people to a different degree and involving not only feelings and the ability to describe them, but also the orientation of thinking of individuals (e.g., external, internal; Taylor, 1984; da Silva et al., 2017).

Indeed, studies so far have shown that individuals with a high level of alexithymia have problems in emotional responses both in terms of emotional regulation and cognitive processing (e.g., Lane et al., 1996; Taylor et al., 1997; Swart et al., 2009). Individuals with a high level of alexithymia have difficulty (i) in perceiving, identifying their feelings and emotions and, in turn, describing them to others; and (ii) incorrectly using emotions either to lead their actions and thinking or to learn from emotional experience (e.g., Luminet et al., 2004, 2006). Recent studies have explained that such difficulties may be due to problems in sensory perception processing (e.g., Serafini et al., 2017). Scholars showed that the inability of alexithymics to identify and describe feelings is associated with a lower response to sensory stimulation and inputs (e.g., Grynberg and Pollatos, 2015; Serafini et al., 2016). Moreover, other studies collected further evidence showing that the lower capacity to recognize emotions can be related to a lower ability in attentional processing (e.g., Vermeulen et al., 2006; Borhani et al., 2016; Correro et al., 2019). In particular, these studies have shown a difficulty of high alexithymia people in the early automatic attentional processing that does not permit to correctly employ their attentional resources for emotional and relevant information in the context (e.g., Aftanas et al., 2003; Swart et al., 2009; Mather and Sutherland, 2011; van der Velde et al., 2015; Nielson and Correro, 2017). Furthermore, a plethora of electrophysiological studies have underlined that the deficit in processing emotional information might be caused by an alteration in the activation of the brain regions (i.e., amygdala, dorsal anterior, and middle cingulate cortex) normally employed by the emotional attention system (e.g., Wager et al., 2008; Diekhof et al., 2011; Pollatos and Gramann, 2011; Van der Velde et al., 2013). Collectively, these difficulties seem to affect, in turn, the memory for emotional information in people with high alexithymia level (Jacob and Hautekeete, 1998; Suslow et al., 2003; Luminet et al., 2006; Vermeulen and Luminet, 2009; Meltzer and Nielson, 2010; Vermeulen et al., 2010, 2018; Donges and Suslow, 2015; Dressaire et al., 2015; Correro et al., 2019; Ridout et al., 2020).

Studies investigating the relationship between alexithymia and memory are increasing in the last years (Jacob and Hautekeete, 1998; Lundh et al., 2002; Luminet et al., 2006; Vermeulen and Luminet, 2009; Correro et al., 2019; Ridout et al., 2020) and are particularly relevant in light of the practical implications that can have, for instance, in the legal arena. Indeed, these studies provided further information to understand whether alexithymia can be one of the several factors that lead to memory distortions and thus make the statements of the people unreliable. However, the results of these studies are contrasting. On the one hand, a few studies have demonstrated that alexithymia does not affect the retrieval of information (e.g., Jacob and Hautekeete, 1998; Lundh et al., 2002). On the other hand, a large number of studies have found a negative impact of alexithymia on memory (e.g., Luminet et al., 2006; Vermeulen and Luminet, 2009; Correro et al., 2019; Ridout et al., 2020). For example, a study by Luminet et al. (2006) showed that people with high alexithymia have reduced recall of emotional information. Specifically, the authors tested the alexithymia of the participants by adopting the typical scale employed for assessing the construct, i.e., the Toronto Alexithymia Scale-20 (TAS-20; Bagby et al., 1994a) consisting of three subscales corresponding to the main features of the construct: (i) DIFs, (ii) DDFs, and (iii) EOT. Then, participants watched a list of emotional (i.e., negative and positive) and neutral words with different instructions to process the information (i.e., perceptual or semantic processing). Finally, the memory of the participants was tested with a remember/know task. Results indicated that high alexithymia participants had a worse recall for the emotional words than low alexithymia participants with regard to the processing adopted during the first phase (i.e., perceptual or semantic) of the study and the type of memory measure (i.e., “remember” or “know”).

In the following study, by adopting a similar procedure, Vermeulen and Luminet (2009) replicated the abovementioned findings. However, the authors added that the impairment in the recollection of the positive and negative words depends on specific subscales of the TAS-20. Indeed, they found that the DIF subscale was the most involved in the lower recall of emotional words in highly alexithymic individuals, while both the DIF and the other subscale EOT were related to the detrimental recall of the positive and negative words in terms of “remember” response.

Further evidence has been provided from another study by Vermeulen et al. (2010) showing that the memory impairment for emotional words in highly alexithymic individuals also depends on the congruency of the emotional valence between the context during the encoding and the studied information. The authors found that the subscales DIF and DDF are associated with the effects on memory when there is congruency between the context and information, while DIF and EOT are related to the recall of the studies information when there is no congruency.

Collectively, all the described studies have provided support to the idea that alexithymia negatively affects memory performance. However, all these studies have demonstrated this issue by using verbal stimuli (i.e., lists of words) instead of more complex stimuli (i.e., pictures, videos). Indeed, only a few studies have tried to fill this gap by adopting pictures of emotional faces or scenes of social interaction (e.g., DiStefano and Koven, 2012; Takahashi et al., 2015). Such studies showed that memory undermining in highly alexithymic individuals also occurs for other types of stimuli. DiStefano and Koven (2012) demonstrated that people with a higher level of alexithymia had a worse recall of the social scenes than people with a lower level of alexithymia. Still, Takahashi et al. (2015) found an association between alexithymia and memory for positive faces but not for the negative ones. Based on these contrasting results as compared to the significant association between alexithymia and negative words, two recent studies by Ridout et al. (2020) further investigated the possibility that alexithymia can also affect the correct recall of complex negative information (i.e., faces and video clips). Results revealed that alexithymia also contributes to the recall of negative faces: The higher the DDF score, the lower the correct recall of negative faces. By contrast, evidence from the second study using emotional video clips demonstrated that alexithymia interferes with the recall of the negative video clip information with regard to the other two subscales of DIF and EOT.

Moreover, recent studies have underlined that memory deficits in highly alexithymic individuals are due to reduced cognitive capacities in terms of executive functioning (EF) (Diamond, 2013). Indeed, high alexithymia people have dysfunction in problem-solving, fluency, shifting, inhibition, and self-report (Henry et al., 2006; Wood and Williams, 2007; Onor et al., 2010; Zhang et al., 2012; Santorelli and Ready, 2015). In particular, some studies have shown that the subscales particularly associated with executive dysfunction are the DIF and DDF (Henry et al., 2006; Koven and Thomas, 2010; Santorelli and Ready, 2015), while other evidence supports the idea that the EOT subscale is the most implicated in the effects of alexithymia on EFs (e.g., Correro et al., 2019).

The idea that the influence of alexithymia on memory is moderated by the EFs abilities can also be supported by studies showing that EFs are essential for cognitive tasks and processes, such as memory recall (Carpenter et al., 2000; Miyake et al., 2000; Duff et al., 2005; Carretti et al., 2010; Hill et al., 2012). In the last years, a plethora of studies have demonstrated that EFs are involved in memory accuracy in terms of both correct information and memory distortions (i.e., omission and commission errors) reported during the retrieval (e.g., Gonsalves and Paller, 2002; Jaschinski and Wentura, 2002; Schacter and Slotnick, 2004; Marsh et al., 2005; Gerrie and Garry, 2007; Peters et al., 2007; Zhu et al., 2010; Leding, 2012; Mirandola et al., 2015; Kersten et al., 2018; Battista et al., 2020). Specifically, it has been shown that the abilities of EF of individuals, in terms of working memory as digit span, shifting as reaction times to different mathematical operations, inhibition as the number of correct responses to visual stimuli, and updating as phonemic fluency, impact on the likelihood to report correct details and errors as follows: Low EF people report less correct details and a higher number of omissions and commissions than high EF people. These recurrent findings have been displayed both for simple stimuli (i.e., words) (e.g., Peters et al., 2007) and for complex stimuli (i.e., video) (e.g., Battista et al., 2020) as well as for neutral stimuli (e.g., Gerrie and Garry, 2007) and emotional stimuli (e.g., Mirandola et al., 2015).

Despite the growing number of studies on alexithymia and memory, research on this topic is still quite limited and findings are often in contrast to each other. Moreover, no studies have verified whether alexithymia is also responsible for a higher or lower susceptibility to report memory distortions (i.e., omissions and commissions). With a forensic perspective in mind, studies investigating a possible relationship among EF resources of individuals, alexithymia, and memory for a complex emotional event, such as a crime scene, seem to be particularly necessary. Thus, this study will try to address this lack of evidence by testing whether the recall of a violent crime (i.e., murder) can be influenced by the three scores of alexithymia (i.e., DIF, DDF, and EOT) in a sample of individuals having high or low EF resources. By doing so, in accordance with previous studies on memory and EFs (Kersten et al., 2018; Battista et al., 2020) as well as Correro et al. (2019) on EF, memory, and alexithymia, we followed a comprehensive approach to measure the EF availability of individuals. In particular, based on studies showing that the three functions of Updating, Shifting, and Inhibition work together while performing cognitive tasks (e.g., recall) (e.g., Kimberg and Farah, 1993; Duncan Roger Johnson Michaela Swales Charles Freer, 1997; Dempster and Corkill, 1999; Miyake et al., 2000), we collapsed in an aggregate measure the individuals' EFs measures of the three EFs. Specifically, Updating refers to the capacity (i) to monitor and code the incoming and relevant information for the execution of a task and (ii) to change the old and irrelevant information into newer and more relevant information (Morris and Jones, 1990; Lehto, 1996). Shifting is the ability to switch among different tasks, operations, or mental sets (Monsell, 1996). Inhibition refers to the capacity to intentionally suppress dominant and automatic responses when necessary (Miyake et al., 2000). Note that the three EFs (i.e., Updating, Shifting, and Inhibition) were chosen also in accordance with recent studies underlining that they are the most implicated in superior cognitive performance, in particular, memory recall (e.g., Espy, 2004; Burgess and Simons, 2005; Diamond, 2013; Kersten et al., 2018; Battista et al., 2020, 2021).

## The Present Study

### Aims and Hypotheses

The current study aimed to verify whether the degree of alexithymia of individuals can influence memory accuracy for a crime experience. More specifically, this study is intended to verify whether the likelihood to recall correct details, memory distortions (i.e., omissions and commissions) for an emotional event is related to the ability to discriminate and describe feelings and the tendency to have EOT. We tested this issue by focusing on memory recall immediately after a target experience and after 10 days. Based on previous evidence showing that alexithymia is related to EF (e.g., Correro et al., 2019) and on studies demonstrating the positive association between EFs and memory (e.g., Peters et al., 2007; Zhu et al., 2010; Leding, 2012; Mirandola et al., 2015; Kersten et al., 2018; Battista et al., 2020), we preliminary assessed the degree of EF resources of participants (i.e., low vs. high) and collapsed the indices of the three EFs of Updating, Shifting, and Inhibition into an aggregate measure. Then, participants filled in some questionnaires measuring their affective state and degree of alexithymia. Immediately after, they watched a mock crime video and answered some questions about it. Ten days later, in a second session, the memory of the participants was assessed for the second occasion. Immediately after, EF resources of the participants were evaluated through three neuropsychological tasks.

In line with previous studies (e.g., Correro et al., 2019), we expected that higher alexithymia scores would be predictive of a lower memory recall, in terms of the amount of correct details (Hypothesis 1). Moreover, in accordance with studies showing that the likelihood to report correct details goes hand in hand with the likelihood to report memory distortions (e.g., Battista et al., 2020), higher alexithymia scores would also be predictive of higher memory distortions (i.e., omissions and commissions) (Hypothesis 2). Finally, based on studies showing that EF influences the effects of alexithymia on memory (e.g., Henry et al., 2006; Koven and Thomas, 2010; Santorelli and Ready, 2015; Correro et al., 2019), we further expected that the negative relationship between alexithymia and memory would be moderated by EF scores of individuals. That is, such a relationship would be stronger in individuals with lower EF resources than in those with higher EF resources (Hypothesis 3).

## Method

### Participants and Design

Using G^*^power (Faul et al., 2007), on a priori power analysis for regression analysis with a power of 0.80, a medium effect size (*f* = 0.15), and four predictors (i.e., TAS-DIF, TAS-DDF, TAS-EOT, and EF) indicated that a sample of 85 participants was required. A sample of 110 undergraduate students (women = 86.4%, *M*_age_ = 21.47, *SD* = 3.11) was thus recruited at the Department of Education, Psychology, Communication of the University of Bari “Aldo Moro.” No specific inclusion and exclusion criteria have been adopted. To achieve the main goal, we created aggregate scores of EF resources (as shown in the section on Results). Therefore, the study was a correlational study with the three scores of alexithymia (i.e., TAS-DIF, TAS-DDF, and TAS-EOT) and EF resources of individuals as predicted variables. The predicted variables were memory scores in terms of correct details and memory distortions (i.e., omissions and commissions scores) in cued recall tests performed after the event (T1) and 10 days later (T2). The ethical committee of the Department of Education, Psychology, Communication of the University of Bari “Aldo Moro” approved the study (No. ET-19-11). Participants did not receive any compensation for taking part in the study and were tested individually in a laboratory. All materials of the study are available on Open Science Framework (OSF): https://osf.io/bwfek/.

### Measures and Procedure

This study consisted of two different sessions. In the first session, participants answered some questionnaires, then watched a video, and answered a cued recall task about the video. The second session was performed after 10 days and required participants to perform the same cued recall and three neuropsychological tasks useful to assess their EF resources. Before starting the experiment, each participant filled in an informed consent form.

### Session 1

Participants were first invited to complete the TAS-20 (Bagby et al., 1994a,b) and the Positive and Negative Affect Schedule-State (PANAS-S; Watson et al., 1988; Terraciano et al., 2003). Thus, participants watched the crime video and finally filled in again the PANAS-S questionnaire to check whether the video emotionally impacted upon them.

**Toronto Alexithymia Scale-20** (Bagby et al., 1994a; Bressi et al., 1996). The TAS-20 consists of 20 5-point items (1 = strongly disagree, 5 = strongly agree). The questionnaire evaluates the level of alexithymia of the participant through three scales: DIF, DDF, and EOT. Specifically, the DIF scale (composed of seven items) measures (Cronbach's α in the present study = 0.85) the proneness of an individual to recognize feelings and emotions and to differentiate them from somatic sensations. The DDF scale (Cronbach's α in the present study = 0.79) assesses the ability of an individual to describe feelings to other people through five items. Finally, the EOT scale (Cronbach's α in the present study = 0.72) consists of eight items measuring the degree of external orientation in the thinking of an individual. Following the scoring system (Bagby et al., 1994a; Caretti et al., 2011), the three subscores are calculated by summing up the answers reported by each participant to the corresponding items of the three scales. Before adding up scores, items 4, 5, 10, 18, and 19 were reversed. The higher the scores, the higher the level of the alexithymia feature of the participant. The score reported by the participants at all the 20 items have been calculated (M = 41.3, SD = 11.8, range 22–77). The total score can vary from 20 to 100 with a cutoff of 61. In this sample, only four people reported a total score higher than 61.

**The PANAS-S** (Watson et al., 1988; Terraciano et al., 2003). The questionnaire assesses the emotional state of participants across two scales: The Positive Affect (PA) and the Negative Affect (NA), composed, in turn, of 10 items. PANAS-S includes 20 5-point items (0 = not at all, 4 = completely). The PA-S (Cronbach's α in the present study = 0.88) and NA-S (Cronbach's α in the present study = 0.89) evaluate the positive and negative affective and emotional state while filling it. The scores of the two dimensions are summed up in accordance with the scoring system (Terraciano et al., 2003). The higher the scores, the higher the affective and emotional state of the participants. Thus, the higher the PA score, the higher the positive emotional state; in addition, the higher the NA score, the higher the negative emotional state of participants.

#### Mock Crime Video

After the completion of the battery of questionnaires, participants were invited to carefully watch a mock crime video. The video (lasting ~2 min 30 s) has been used in other memory studies (Mangiulli et al., 2019; Battista et al., 2020) and shows a debate between two men. This discussion continues with a fight between the two men and the murder of one by the other. Having seen the video, participants filled the PANAS-S (Watson et al., 1988; Terraciano et al., 2003).

#### Memory Test Phase (T1)

Participants completed a memory test by imagining to be an eyewitness of the crime and to provide testimony to a police officer. The test was a cued recall task; hence, participants answered 14 open questions (i.e., *What was the victim wearing?*) based on what they remember having seen during the video and without guessing. The questions asked participants to recall several details of the crime scene in the video (i.e., the weapon of the murder, the wearing of the murderer and victim, etc.) and are reported in Appendix A (Supplementary Material). All the questions were proposed in the same order to all participants. This memory test has been already adopted in prior studies on memory (e.g., Battista et al., 2020).

### Session 2

#### Memory Test Phase (T2)

After 10 days, participants came back to the laboratory and answered the same questions of the cued recall performed at Session 1. The questions were proposed in the same order of the cued recall administered at T1 to all participants.

### Executive Functioning Assessment

After the memory test, participants completed the three neuropsychological tasks assessing their EF resources. The first two were paper and pencil tasks, while the third one was a computerized task of the Psychology Experiment Building Language 2.0 battery (PEBL 2.0; Mueller and Piper, 2014). At the end of the three tasks, participants were thanked and debriefed.

**The phonemic fluency** (Novelli et al., 1986). The task measures the ability of Updating (Miyake et al., 2000). The experimenter gives a letter and participants have to refer to as many words as possible in 60 s, beginning with such a letter and excluding names of people and cities. The experiment provides three different letters (C, P, and S). The score of this task is the average number of words produced for each letter. The score is computed by excluding repetitions (i.e., words repeated more times) and intrusions (i.e., words not conforming with the instructions).

**Plus-minus task** (Jersild, 1927; Spector and Biederman, 1976). This task assesses the capacity of Shifting (Miyake et al., 2000). It is composed of three trials of mathematical operations (i.e., additions and subtractions) on three different sets of random numbers. Specifically, in the first trial, participants have to add the value of three to each number, while in the second trial, they have to subtract three from each number. In the final trial, participants have to alternate among additions and subtractions, that is, participants start with adding three to the first number, continue with subtracting three to the second number, and proceed by switching between the two operations. The experimenter records the time to perform the three trials. The score is calculated by subtracting the average time of the first two trials from the time of the final trial.

**Go-NoGo task** (Bezdjian et al., 2009). The task measures the ability of Inhibition (Miyake et al., 2000). The task is carried out by using a computer, where participants watch a square divided, in turn, into four squares where the letter P or R appears in a random fashion. Participants are instructed to respond to some stimuli (Go stimuli) and give no response to others (NoGo stimuli) based on the block they are performing. During the first block, the P-Go block, participants have to press the mouse every time the P appears on the screen and not press when the letter R appears. During the second block, the R-Go block, participants have the opposite instructions: Press the mouse when the letter R appears and not press when the letter P appears on the screen. Both blocks are comprised of 160 stimuli and the letters appear on the screen for 500 ms. The score is the commission errors reported at the two blocks.

### Cued Recall Scoring

The scoring of both the cued recall tasks (i.e., T1 and T2) was independently performed by the first author and a student assistant, blind to the experimental design. The scoring followed a scoring system employed in a prior study (e.g., Battista et al., 2020). Thus, the three scores of correct details, omissions, and commissions were computed as follows. With regard to the correct details score, one point was assigned for each completely correct answer (e.g., “*What was the victim wearing?” “He was wearing a black jacket, white skirt, and black shirt”*), half a point was assigned for a partially correct answer (e.g., “*He was wearing a black jacket”*), and zero was given for no answer (e.g., “*I do not remember”*) or a completely wrong answer (e.g., “*He was wearing a green suit”*). Regarding the omissions score, one point was given when no answer was provided (e.g., “*I do not remember”*). Concerning the score of the commission, one point was assigned when new and wrong (i.e., not present in the video) information was reported (e.g., “*He was wearing a green suit”*) and half a point when a partially distorted answer was provided (e.g., “*He was wearing a blue jacket, blue skirt, and black shirt”*). The maximum score obtainable for the three indices was 14. In addition, proportions were calculated by dividing the score obtained by each participant by the maximum score obtainable (i.e., 14). The ICC average measure for the number of correct details at T1 and T2 was good: 0.77 and 0.68 (both *ps* < 0.001), respectively. The ICC average measure of omissions and commissions was also good: 0.79 and 0.78 (both *ps* < 0.001) for T1 and 0.78 and 0.76 (both *ps* < 0.001) for T2.

### Data Analysis

First, we calculated an aggregate measure of EF for each participant. To order and compare the resources of individuals for the three EFs based on the performance reported at the tasks, we first calculated the rank distributions for each score and then calculated the average of the rank scores. We followed this approach in accordance with previous studies on memory and EFs (e.g., Kersten et al., 2018; Battista et al., 2020). Then, to check the emotional impact of the mock crime video on the affective state of the participants, two paired samples *t-*tests were run on the scores of the two subscales of PANAS-S (i.e., PA and NA). Finally, to verify the Hypothesis 1, 2, and 3 and after checking the associations among the memory scores, the three TAS scores, and EF score (i.e., Pearson's correlation), a series of general linear model (GLM) analyses were run on the memory scores for correct details (Hypotheses 1), omissions, and commissions (Hypothesis 2) both at T1 and T2. In such analyses, we tested the main effects of the alexithymia scores (i.e., TAS-DIS, TAS-DDF, and TAS-EOT) and the EF score as well as their interaction effects. Moreover, when we found a significance for the interaction effects, we carried out moderation analyses considering the EF score as a moderator (Hypothesis 3). All the analyses have been computed by using *Jamovi* 1.1.8 (Love et al., 2021) that adopts the Preacher and Hayes approach (Preacher and Hayes, 2004).

## Results

### Analyses on the Aggregate Measure of EF

We created the aggregate measure of EF as follows. First, we checked the rank distributions for each neuropsychological task (i.e., Phonemic Fluency, Plus-Minus Task, and Go/NoGo). Then, we calculated the aggregate score for each participant by averaging the rank score across the three tasks (Table 1).

**Table 1:** Table shows the mean reported by participants at the three EF (i.e., Shifting, Inhibition, and Updating) tasks and the aggregate score of EFs.

**EFs scores**	
Shifting	85.98 (32.44)
	95% CI [79.85 – 92.11]
Inhibition	309.38 (32.44)
	95% CI [396.38 – 312.39]
Updating	14.80 (3.35)
	95% CI [14.16 – 14.85]
Aggregate score	19.58 (13.07)
	95% CI [17.11 – 22.05]

*In particular, the Shifting score refers to the time employed in seconds, the Inhibition score is the average of correct responses, and the Updating score is the average of words reported. Standard deviations and 95% CI are shown between parentheses*.

### Impact of the Crime Video

In order to assess the emotional impact of the mock crime on participants, a paired samples *t-*test with mock crime (pre vs. post) as a *within-factor* was run on PA-S and NA-S scores. The analysis showed a statistically significant difference on PA-S, *t*_(109)_ = 3.62, *p* < 0.001, *d* = 0.35. In particular, the positive state was significantly lower after the video than before it, *M*_pre−mock crime_ = 25.69, 95% CI [24.39, 26.99] vs. *M*_post−mock crime_ = 23.58, 95% CI [22.15, 25.02]. By contrast, no statistically significant difference was found on NA-S, *t*_(109)_ = −0.59, *p* = 0.56, *d* = −0.06. Although no statistical significance was reached, the negative state appeared to increase after the video presentation (*M*_pre−mock crime_ = 6.48, 95% CI [5.00, 7.97] vs. *M*_post−mock crime_ = 6.77, 95% CI [5.35, 8.19]).

Moreover, with the aim to verify whether the affective (i.e., positive and negative) states of participants were related to their alexithymia level, Pearson's correlations were carried out among the PA-S and NA-S scores (pre- and post-mock crime) and the three TAS scores (i.e., TAS-DIF, TAS-DDF, and TAS-EOT). We found that TAS-DIF was positively associated with NA-S scores, both pre- and post-mock crime, *r* = 0.44, *p* < 0.001 and *r* = 0.36, *p* < 0.001, respectively. Moreover, TAS-DDF negatively correlated with PA-S scores, both pre- and post-mock crime, *r* = −0.24, *p* = 0.01 and *r* = 0.20, *p* = 0.04, respectively, as well as positively correlated with NA-S scores, both pre- and post-mock crime, *r* = 0.38, *p* < 0.001 and *r* = 0.27, *p* = 0.004, respectively. Finally, TAS-EOT was only negatively associated with the PA-S score pre-mock crime, *r* = −0.25, *p* = 0.008. No other statistically significant correlations were found (*p* > 0.05).

### Executive Functioning and Alexithymia

To verify whether the EF resources of individuals are related to alexithymia, Pearson's correlations were run between the three TAS-20 scores (i.e., TAS-DIF, TAS-DDF, and TAS-EOT) and the EF score. Only TAS-DIF correlated significantly with EF, *r* = 0.27, *p* = 0.005. By contrast, no statistically significant correlation was found between TAS-DDT and TAS-EOT with EF, *r* = 0.17, *p* = 0.07. and *r* = 0.05, *p* = 0.64, respectively.

### Cued Recall Scores^1^

^1^

Pearson's correlations were run among the three TAS-20 scores (i.e., TAS-DIF, TAS-DDF, and TAS-EOT), the EF score, and memory scores (i.e., correct details, omissions, and commissions at T1 and T2). All the correlation indices are shown in Table 2. Regarding alexithymia, only TAS-EOT correlated significantly with omissions score at T2, *r* = −0.32, *p* < 0.001. No other statistically significant correlation was found between alexithymia and memory scores (i.e., correct details, omissions, and commissions at T1 and T2) (*p* > 0.05). Moreover, the EF score was statistically significant correlated with correct details, omissions, and commissions scores at T1, *r* = 0.55, *p* = 0.05, *r* = −0.30, *p* = 0.002, and *r* = −0.28, *p* = 0.004, respectively.

**Table 2:** Table shows Pearson's correlation scores among TAS scores, the EF score and memory scores (i.e., correct details, omissions, and commissions) both at T1 and T2.

	**TAS-DIF**	**TAS-DDF**	**TAS-EOT**	**EF score**
Correct details T1	0.04	−0.03	−0.10	0.55^*^
Correct details T2	0.12	−0.08	−0.01	0.04
Omissions T1	−0.06	0.01	0.13	−0.30^*^
Omissions T2	0.06	0.02	−0.32^**^	0.03
Commissions T1	0.03	−0.04	0.07	−0.28^*^
Commissions T2	−0.08	0.04	0.08	−0.04

* *p < 0.01*,

** *p < 0.001*.

A set of GLM with the three TAS scores (i.e., TAS-DIF, TAS-DDF, and TAS-EOT) and EF scores as predictors was carried out on the cued recall scores of correct details, omissions, and commissions both at T1 and at T2 (i.e., predicted variables). All the direct and the interaction effects are reported in Table 3.

**Table 3:** Table shows the direct and interaction of effects tested in our General Linear Models on the memory scores.

			***β, t, p***			
	**TAS-DIF**	**TAS-DDF**	**TAS_EOT**	TAS-DIF*EF	TAS-DDF*EF	TAS-EOT*EF
Correct details T1	0.11,0.80,0.42	−0.09, −0.61, 0.54	−0.18, −1.55, 0.12	−0.08, −0.28, 0.78	0.02, 0.07, 0.94	−0.44, −1.93, 0.56
Correct details T2	0.15, 1.06, 0.29	−0.04, −0.26, 0.80	−0.03, −0.27, 0.79	0.16, 0.57, 0.57	0.03, 0.10, 0.92	−0.47, −2.11, 0.04
Omissions T1	−0.09, −0.66, 0.51	0.16, 1.13, 0.26	−1.17, −1.54, 0.13	−0.07, −0.25, 0.81	0.11, 0.39, 0.70	0.15, 0.68, 0.50
Omissions T2	0.03, 0.22, 0.83	0.10, 0.70, 0.49	−0.39, −3.66, <0.001	0.29, 1.49, 0.30	−0.29, −1.00, 0.32	0.16, 0.75, 0.46
Commissions T1	0.01, 0.07, 0.94	−0.11, −0.75, 0.46	0.05, 0.46, 0.65	0.14, 0.50, 0.62	−0.14, −0.49, 0.63	0.18, 0.81, 0.42
Commissions T2	−0.13, −0.91, 0.37	0.05, 0.35, 0.72	0.02, 0.19, 0.85	−0.12, −0.43, 0.67	<0.001, 0.003, 0.99	−0.57, −2.56, 0.01

#### Correct Details

With regard to the correct details score, none of the TAS scores predicted the likelihood to report correct details at T1 as well as no interaction effects among the three TAS scores and EF was significant (*p* > 0.05). Overall, the model fit indices indicated an acceptable fit of the model (Burnham and Anderson, 2002), *R*^2^ = 0.05, RMSE = 0.067, *F*_(2, 102)_ = 0.83, *p* = 0.56, η^2^ = 0.05.

Regarding the correct details score at T2, all the main effects of TAS scores were not statistically significant. However, the interaction effect of EOT with EF reached the significance level, β = −0.47, *t* = −2.11, *p* = 0.04. Simple slopes analyses (i.e., moderation analyses) showed that, as long as the EF aggregate score decreases, EOT scores are negatively associated with the proportion of correct details reported at T2, β = −0.004, *t* = −1.65, *p* = 0.04. No other interaction effects were statistically significant (*p* > 0.05). The model fit indices were *R*^2^ = 0.07, RMSE = 0.076, *F*_(2, 102)_ = 1.07, *p* = 0.39, η^2^ = 0.07, showing that the model had an acceptable fit (Burnham and Anderson, 2002).

#### Omissions

Concerning the omissions score at T1, none of the three TAS scores were found associated with the likelihood to report omissions at T1 (*p* > 0.05). The interaction effects between EF and the three TAS scores were not statistically significant (*p* > 0.05). The model reached the following fit indices: *R*^2^ = 0.08, RMSE = 0.092, *F*_(2, 102)_ = 1.24, *p* = 0.29, η^2^ = 0.08, suggesting that the model did not have a good fit (Burnham and Anderson, 2002).

With regard to the omissions score at T2, the main effect of TAS-EOT was statistically significant, β = −0.39, *t* = −3.66, *p* < 0.001. That is, the higher the EOT score, the lower the omissions at T2. In contrast, the direct effects of TAS-DIF and TAS-DDF did not reach statistical significance (*p* > 0.05). In addition, the interaction effects between EF and the three TAS scores were not statistically significant (*p* > 0.05). The model fit indices were *R*^2^ = 0.13, RMSE = 0.104, *F*_(2, 102)_ = 2.16, *p* = 0.04, η^2^ = 0.13, indicating that the model did not have a good fit (Burnham and Anderson, 2002).

#### Commissions

Regarding the commissions score at T1, none of the three TAS scores predicted the likelihood to report commissions at T1 as well as the interaction effects (*p* > 0.05). The model fit indices were acceptable, *R*^2^ = 0.07, RMSE = 0.082, *F*_(2, 102)_ = 1.20, *p* = 0.31, η^2^ = 0.08.

Finally, concerning the commissions score at T2, none of the main effects of TAS scores was statistically significant (*p* > 0.05). The interaction effect EF^*^EOT was statistically significant, β = −0.57, *t* = −2.56, *p* = 0.01. Simple slopes analyses (i.e., moderation analyses) showed that as EF score decreases, a negative relationship between EOT and commissions at T2 becomes significant, β = −0.01, *t* = −2.46, *p* = 0.02. The other interaction effects of EF and TAS subscales were not statistically significant (*p* > 0.05). The model reached good fit indices *R*^2^ = 0.09, RMSE = 0.077, *F*_(2, 102)_ = 1.49, *p* = 0.18, and η^2^ = 0.09 (Tables 3, 4).

**Table 4:** Mean proportions of the memory scores (i.e., correct details, omissions, commissions) reported during the first (T1) and the second (T2) memory test by participants.

**Memory scores**	**T1**	**T2**
Correct details	0.37 (0.07)	0.41 (0.08)
	95% CI [0.36, 0.38]	95% CI [0.40, 0.43]
Omissions	0.40 (0.10)	0.34 (0.11)
	95% CI [0.38, 0.41]	95% CI [0.32, 0.36]
Commissions	0.23 (0.09)	0.58 (0.08)
	95% CI [0.22, 0.25]	95% CI [0.57, 0.60]

*Standard deviations and 95% CI are shown between parentheses*.

## Discussion

The current study aimed to verify the role of alexithymia on memory accuracy for a violent crime. Collectively and in accordance with prior studies (e.g., Vermeulen et al., 2010; Correro et al., 2019; Ridout et al., 2020), we found that a higher alexithymia level (only in terms of EOT score) is associated with lower correct details and higher memory distortions reported by participants during the recall of the event, especially with regard to the EF resources of individuals. That is, the lower the EF, the higher the negative effect of TAS on memory performance. By contrast, we did not find a similar pattern of results for the DIF and DDF scores.

### Alexithymia, Correct Details, and EF

To begin with, we demonstrated that the EF resources of individuals are positively associated with the proportion of correct details at T1. The higher the EF resources, the higher the proportion of correct details reported by participants at T1. This is completely in accordance with studies conducted so far showing that having high EF resources facilitates the encoding of an event by resulting in a better retrieval (e.g., Battista et al., 2020). Due to their availability of EF resources, high EF people are more able to shift among the details of an event (i.e., Shifting), suppress interferences and irrelevant information (i.e., Inhibition), and monitor relevant information (i.e., Updating) at the encoding than low EF people. Having a better encoding of original material allows them to better retrieve that material. In contrast with the expectation (Hypothesis 1), the analysis showed no main effect of the three scores of alexithymia (i.e., TAS-DIF, TAS-DDF, and TAS-EOT) on the recollection of correct details at both T1 and T2. This is also in contrast with the results achieved by previous studies (e.g., Vermeulen et al., 2010; DiStefano and Koven, 2012; Takahashi et al., 2015; Correro et al., 2019; Ridout et al., 2020). For example, Ridout et al. (2020) found that at least the ability to identify and interpret feelings and emotions (i.e., DIF) is negatively associated with the correct recall of emotional information for complex stimuli, such as short video clips. The authors stressed the idea that the inability to correctly process emotional information during the encoding results in a worse retrieval of such information. Probably, the lack of evidence can be explained by considering the type of stimuli adopted. Indeed, in this study, we used a video of a mock crime showing the murder following a quarrel between two people. Although the analyses confirmed that the video emotionally affected the emotional states of the participants by decreasing the initial positive state, we did not find an increase in the negative states of the participants after watching the video. Therefore, it can be that we failed to replicate a direct effect of alexithymia scores on memory recall because the video partially impacted the emotional states of the participants. Although there was no main effect of the three alexithymia scores, the results demonstrated that the interaction between EOT (i.e., the characteristic of directing thoughts at external reality and hardly or not at inner experience) and EFs affects the correct recall of the details of the event at T2. Indeed, we found that the negative effect of EOT on the proportion of correct details recalled at T2 increased with a decrease in the availability of EF resources of an individual. Although some scholars have pointed out that EOT is not a proper representative of the characteristic of alexithymia (e.g., Kooiman et al., 2002), the results underline the idea that EOT, because it is the characteristic of alexithymia that impedes people to have internal monitoring (e.g., Correro et al., 2019), does not allow the use of internal cognitive control that promotes memory processes (Dressaire et al., 2015). Moreover, in line with the Hypothesis 3 for which we expected that alexithymia would have affected the recall by interacting with the availability of EF resources of an individual, we found that the negative association between having an externally oriented style of thinking and the amount of correct details reported is moderated by the availability of EF resources of an individual. These findings are in accordance with previous findings (e.g., Henry et al., 2006; Correro et al., 2019) for two reasons. First, we confirm that EOT seems to be the main characteristic of alexithymia that influences memory performance instead of the abilities to identify and describe feelings and emotions (i.e., DIF and DDF). Indeed, in a recent study and across three experiments, Correro et al. (2019) have shown that the negative effects of alexithymia on the encoding and, in turn, on the retrieval of information can depend on EOT. Second, we provided further support to the idea that such a negative influence of alexithymia on memory is strictly linked to the EFs of the individuals (e.g., Henry et al., 2006; Correro et al., 2019; Ridout et al., 2020). Alexithymia does not directly affect memory performance but only when interacts with the EF resources of individuals. This is also in line with studies showing that alexithymia is associated with executive dysfunction (e.g., Henry et al., 2006; Correro et al., 2019; Ridout et al., 2020). As a matter of fact, in the screening analyses, we also found that a higher amount of alexithymia is associated with lower EF resources. We found this negative association with regard to the DIF score rather than the EOT score. Note that although we found a statistically significant interaction effect between EF and EOT on correct details score, the findings of the screening analysis are fully concordant with prior studies underlying the negative association between EF and alexithymia (i.e., DIF) (e.g., Henry et al., 2006; Correro et al., 2019).

### Alexithymia, Memory Distortions, and EF

The findings on memory distortions (i.e., omissions and commissions) stressed an interesting pattern of results. We first found that the likeliness to report omissions (i.e., details encoded but not recalled) at T1 is negatively associated with the EF score. Thus, the lower the EF score, the higher the omissions at T1. This finding confirmed previous research showing that low EF individuals are more prone to report memory distortions than high EF individuals (e.g., Battista et al., 2020). However, we did not find a similar pattern of results with regard to the omissions reported at T2. This seems to suggest that EF abilities influence the proneness to report this kind of distortions only immediately after the event. A possible explanation could be that the differences in cognition of people, that typically inform on the formation of memory distortions, are downsized by the delay in the recollection, and this leads to similar memory performance. In addition, the data showed that the likelihood to report this type of distortion is related to the main effect of the EOT score. Indeed, we also found a significant direct effect of EOT on omissions scores at T2: The higher EOT, the lower the omissions regardless of the EF availability of the individuals. This evidence is in contrast with Hypothesis 2: We found an effect of alexithymia score (i.e., EOT) but in a contrasting direction. The results suggest that a higher tendency to EOT leads to report fewer omissions at T2. That is, participants with a high level of EOT are able to recall more event-related details than participants with a low level of EOT. However, so far, scholars have argued that EOT interferes with the encoding of information by resulting in a lower recall of information (e.g., Correro et al., 2019). This makes it reasonable to believe that this inability to correctly encode information would lead to reporting lower information during the retrieval. However, the finding seems to provide two important insights: (i) The influence of the EOT level of individuals depend on when people recall the event-related information and (ii) people with high EOT level report more information (i.e., fewer omissions) during a late recall than people with low EOT level. A possible explanation can be that EOT negatively impacts the encoding and, in turn, the retrieval in terms of the correctness (i.e., true vs. distorted or false) of the information reported rather than in the amount of information recalled. The findings seem to support the idea that having EOT does not lead to forgetting of the experienced information (e.g., Vermeulen and Luminet, 2009; Ridout et al., 2020).

The pattern of results concerning the commission score can be explained as follows. We found that EF resources are also associated with the amount of commissions (i.e., details distorted or false with respect to the details encoded) at T1. Again, we demonstrated that the lower the EF score, the higher the commissions reported at T1. This is in accordance with prior studies demonstrating that a high amount of distorted information or information never encoded is due to lower EF resources (i.e., Mirandola et al., 2015; Battista et al., 2020). In addition, this result perfectly fits with the data of the current study on the correct details and with prior evidence showing that the likelihood to report more commissions goes hand in hand with the likelihood to report lower correct details (e.g., Battista et al., 2020). Contrary to our expectations, no statistically significant main effect of alexithymia (i.e., TAS-DIF, TAS-DDF, and TAS-EOT) was found for the commissions reported at both T1 and T2. However, we showed that EOT score significantly interacted with EF resources on the score of the commission at T2. Specifically, we demonstrated that the EF resources of individuals moderate the effect of EOT on commissions recalled at T2: As EF decreases, the negative association between EOT and commissions becomes statistically significant. Again, this is in line with the findings on the correct details score at T2 and Hypothesis 3. Combining the explanations provided by the line of research on EF and memory distortions (e.g., Gerrie and Garry, 2007; Peters et al., 2007; Unsworth and Brewer, 2010; Leding, 2012; Battista et al., 2020) and the second line on alexithymia and memory (e.g., Correro et al., 2019; Ridout et al., 2020), we can explain this latter finding as follows. In general, having lower EF resources makes people more susceptible to encode interferences and less focused on the relevant event-related information during the encoding. This, in turn, makes it difficult to distinguish the original information from the one imagined during the retrieval resulting in more commissions than high EF individuals (e.g., Roediger and McDermott, 1995). Moreover, scholars have been argued that a higher external-oriented style of thinking (i.e., EOT) does not permit to employ the necessary cognitive resources to correctly encode incoming information (e.g., Correro et al., 2019). Hence, the EF resources of individuals further affect the normal bad encoding of participants with high EOT by making people more likely to report commissions.

### Limitations

Although the study has the merit to provide new evidence on unexplored questions, such as the role of alexithymia on the formation of memory distortions, some limitations need to be considered. For instance, the study was correlational and, thus, although we have provided information on the relationship between alexithymia and memory, we were not able to provide a cause-and-effect direction between the variables of interest. Furthermore, the composition of the sample could have underestimated the results. Indeed, we tested students with a small range of age and, thus, similar EF. The findings could be not representative of what occurs in the normal population. Moreover, related to this first limitation, it could be the case that the investigation of the relationship between alexithymia and EF might be different in a sample composed of people categorized as alexithymic (i.e., individuals reporting a level of alexithymia higher than the cutoff). Another limitation is due to the emotional stimulus adopted. We indeed wanted to test how alexithymia interferes in the recall of an emotional and complex stimulus. We thus administered to participants a video of mock violent crime that showed in previous research an emotional impact on the state of the participants. However, in the present experiment, the video partially impacted the state of the participants. Hence, it is possible that different findings could occur for a stronger emotional stimulus. Moreover, because the primary aim of our study was to replicate a real forensic situation as much as possible, we did not include a non-emotional stimulus. Nevertheless, including a control situation (i.e., non-emotional video) could further inform on the relationship between alexithymia and the ability to recall an experience. Further studies are necessary to gap these caveats.

## General Conclusion and Practical Implications

In conclusion, the present experiment provided further information on the relationship between alexithymia, EF, and memory. A high degree of alexithymia (i.e., EOT score) leads to negative mnemonic effects with regard to both correct details and memory distortions, and this depends also on the availability of EF of an individual. Our findings are particularly helpful for legal professionals that deal with the memory-related statements of individuals in legal proceedings. Collectively, the results suggest that alexithymia can contribute to the formation of memory distortions; hence, it is an important factor to take into consideration for the assessment of the reliability of witnesses, suspects, and the statements of victims. Legal professionals have to keep in mind that participants with a high level of alexithymia or people with a diagnosis of alexithymia might unintentionally provide unreliable information. This is particularly relevant within the legal arena as statements represent one of the main sources for legal practitioners (e.g., judges) to correctly perform their job and that can lead to accurate or wrong legal decisions (e.g., conviction, absolution). In addition, on a more general note, the findings of the current study can be relevant also for clinical psychologists that work with alexithymic patients. Indeed, such findings can inform them of the (un)ability of patients to correctly retrieve emotional experiences useful to structure a clinical intervention.

## Data Availability Statement

The raw data supporting the conclusions of this article will be made available by the authors, without undue reservation.

## Ethics Statement

The studies involving human participants were reviewed and approved by Department of Education, Psychology, Communication of the University of Bari Aldo Moro. The patients/participants provided their written informed consent to participate in this study.

## Author Contributions

FB conceived the study, conducted data collection, analyze the data, and wrote the manuscript. TL and AC critically revised the manuscript. All authors contributed to the article and approved the submitted version.

## Conflict of Interest

The authors declare that the research was conducted in the absence of any commercial or financial relationships that could be construed as a potential conflict of interest.

## Footnotes

^1^We also run exploratory analyses considering the three measures of EFs separately. The analyses showed a statistically significant interaction effect of Shifting^*^DIF on the amount of correct details and commissions reported at T2. In addition, also the interaction effect of Shifting^*^EOT on the amount of commissions reported at T2 was statistically significant. Specifically, the results demonstrated that as long as the Shifting score decreases, DIF is positively associated with correct details score and negatively associated with the amount of commissions at T2. Similarly, as long as the Shifting score decreases, EOT is negatively associated with the amount of commissions at T2. These analyses can be found on: https://osf.io/bwfek/.
