# Supplementary material for: Does Alexithymia Affect Memory for a Crime? The Relationship Between Alexithymia, Executive Functions, and Memories
Source: Front Psychol. 2021 Jun 30;12:669778. doi: 10.3389/fpsyg.2021.669778 (PMC8278017; doi:10.3389/fpsyg.2021.669778)
Supplement: Supplementary file 1 [file Data_Sheet_1.docx]

**Appendix A**

1. *Where did the murder happen?*
2. *What was the victim wearing?*
3. *How many gunshots were fired?*
4. *Were the wall of the bathroom coloured? If so, what colour?*
5. *Do you remember some blood on the scene? If so, where?*
6. *Was the victim armed? If so, what weapon did they have?*
7. *What did the murderer use to kill?*
8. *Was the murderer armed? If so, what weapon did they have?*
9. *In the room where the body was found there was a sink. What objects were on the edge?*
10. *Was the victim wearing accessories? If so, which ones?*
11. *Did the victim try to defend themselves? If so, how?*
12. *What was the murderer wearing?*
13. *Do you remember the presence of any objects in the garage?*
14. *When did the victim die?*

Appendix A reports the 14 questions of the cued recall used to assess participants’ memory at T1 and T2.
